# The association between experiences of racism and mental health on children and young people in the UK: rapid scoping review

**DOI:** 10.1192/bjo.2024.836

**Published:** 2025-01-27

**Authors:** Fiyory Tzeggai Ghezae, Zonke Zungu, Ann John, Kadra Abdinasir, Kamaldeep Bhui, Adenike Adebiyi, Cathy Creswell

**Affiliations:** Department of Experimental Psychology, University of Oxford, Oxford, UK; and Faculty of Health and Life Sciences, Oxford Brookes University, Oxford, UK; Section of Organisational Psychology, University of Cape Town, Rondebosch, South Africa; Swansea University Medical School, Swansea, UK; Centre for Mental Health, London, UK; Department of Psychiatry, University of Oxford, Oxford, UK; Academic Psychiatry, University of Oxford, Oxford, UK; Department of Experimental Psychology, University of Oxford, Oxford, UK

**Keywords:** Childhood experience, stigma and discrimination, social functioning, education and training, clinical outcomes measures

## Abstract

**Background:**

Racism is increasingly recognised as a key contributor to poor mental health. However, the existing literature primarily focuses on its effects on adults.

**Aim:**

To identify literature on the association between experiences of racism and mental health in children and young people in the UK.

**Method:**

Inclusion criteria were: (a) peer-reviewed publications containing original data; (b) UK-based research; (c) included examination of associations between mental health and experiences of direct or indirect racism (quantitative or qualitative); (d) inclusion of an assessment of mental health outcomes; (e) participant ages up to and including 18 years of age or (if the range went beyond 18) with a mean age of 17 years or less. Six databases were searched between 2000 and 2022; an initial 11 522 studies were identified with only eight meeting the inclusion criteria.

**Results:**

Five of the identified studies provided quantitative data and three provided qualitative data. The majority of studies (7/8) focused on children and young people aged 10 years and over; only one focused on children under the age of 10 years. Measurements of racism varied among the studies providing quantitative data. Only four studies directly focused on the effects of racism on the mental health of children and young people.

**Conclusion:**

Although the included studies highlighted potential negative impacts of experiences of racism on children and young people in the UK, this review shows the lack of available literature to inform policy and practice. No studies examined the impact of internalised racism, systemic and institutional racism, or intersectionality.

In light of recent high profile racially motivated incidents, both globally and specifically in the UK, the impact of racism on health and well-being has become the subject of media attention as well as research, policy and practice. For example, in the UK, a surge of instances of racist abuse posted on social media ensued following the 2016 referendum about the UK's membership of the EU (‘Brexit’)^1^ and the 2019 death by drowning of 12-year-old Shukri Yahye-Abdi – a young, Black Muslim schoolgirl – sparked protest as many suspected racial motivation behind the bullying in the lead up to her death. The 2020 murder of George Floyd, an unarmed Black man, by a White police officer in the USA sparked global protest.

In this paper, we refer to various types of racism, such as internalised, interpersonal, institutional and structural racism. Definitions are provided in Table 1, taken from USA-based racial justice organisation Race Forward (https://www.raceforward.org/). We also refer to vicarious racism using a recommended definition.^2^

**Table 1:** Definitions for racism

Individual racism	Definition
Internalised racism lies *within individuals*	‘These are private beliefs and biases about race that reside inside our own minds and bodies. For White people, this can be internalized privilege, entitlement, and superiority; for people of color, this can be internalized oppression. Examples: prejudice, xenophobia, conscious and unconscious bias about race, influenced by the White supremacy.’
Interpersonal racism occurs *between individuals*	‘Bias, bigotry, and discrimination based on race. Once we bring our private beliefs about race into our interactions with others, we are now in the interpersonal realm. Examples: public expressions of prejudice and hate, microaggressions, bias and bigotry between individuals.’
Vicarious racism^2^	‘The second-hand exposure to the racial discrimination and/or prejudice directed at another individual.’
Institutional racism occurs *within institutions*	‘It involves unjust policies, practices, procedures, and outcomes that work better for White people than people of color, whether intentional or not. Example: A school district that concentrates students of color in the most overcrowded, under-funded schools with the least experienced teachers.’
Structural racism is racial inequities *across* institutions, policies, social structures, history and culture	‘Structural racism highlights how racism operates as a system of power with multiple interconnected, reinforcing, and self-perpetuating components which result in racial inequities across all indicators for success. Structural racism is the racial inequity that is deeply rooted and embedded in our history and culture and our economic, political, and legal systems. Examples: The‘racial wealth gap,’ where Whites have many times the wealth of people of color, resulting from the history and current reality of institutional racism in multiple systems.’

All quotes are definitions taken from Race Forward.

Notwithstanding the usefulness of these definitions, the literature suggests the use of the terms ‘racism’ and ‘racial discrimination’ is interchangeable.^3^ Both are used in reference to unequal and harmful distribution of power in societies on the basis of cultural, ancestral or phenomenological difference among individuals.^3^ Indeed, when considering racism definitions have related interchangeably to discrimination relating to individuals’ ‘race’, ‘ethnicity’ and ‘ancestry’.^4^ Whereas race is a sociopolitical construct that groups people based on perceived physical traits, ethnicity captures elements of an individual's identity beyond physical characteristics, such as culture, language and religion.^5^ Ancestry can be further broken down into geographical, genealogical and genetic characterisations.^5^

Although racism is increasingly recognised as a key contributor to poor mental health,^6^ the existing literature primarily focuses on the effects of racism on adults and/or is based in the USA (e.g. Heard-Garris et al^2^ and Paradies et al^6^). A number of meta-analyses found a strong association between poor mental health outcomes and racial discrimination in both adults,^7,9^ and among children and adolescents.^10^ For example, a significant association between racism and suicidality was found in a systematic review of studies of young people in the USA.^11^ Studies on Indigenous Australians^12^ and ethnic minority groups based in the UK^13^ are fewer but have also indicated associations between experiences of racism and poor mental health. For example, Chakraborty et al^13^ found that there was an association between experiences of racial insults and higher scores on a psychosis screening questionnaire among participants aged 16–74 years who were Caribbean, Bangladeshi and Pakistani in England. The experiences that young people have of racism varies. They may experience interpersonal racism, or they may notice the impact on their parents, families and communities through vicarious racism. They may not necessarily connect their experience of exclusion, marginalisation or bullying to race, culture or ethnicity; conversely, they may be too aware, potentially resulting in internalised racism.^14^ What is clear is that direct and vicarious experiences of racism can result in race-based traumatic stress; that is, the psychological and physiological impact of exposure to racial discrimination, which can be debilitating if prolonged.^15^ Racial trauma may explain the disproportionate prevalence of exposure to trauma among children and young people of colour compared with White children and young people.^16^ Moreover, it is suggested that in comparison to general bullying, race-based bullying, a source of racial trauma, has been associated with more negative mental health^17^ and general health consequences.^17^

National surveys are important for providing data on the mental health of minoritised ethnic groups, which may give some indication of the effects of experiences of racism. Data with adult populations in England suggest that common mental health disorders, post-traumatic stress and psychoses are more prevalent in adults from minoritised groups than in ‘White’ adults, with particularly high rates among ‘Black’ adults.^18^ (We note that both ‘White’ and ‘Black’ have been put in quotations to express the imprecisions of racial categories.) It is well established that many of these disorders commonly first have their onset in childhood or adolescence (e.g. Solmi et al^19^). However, in contrast, recent NHS data suggest ‘White’ children and young people are *more* likely to have a mental health condition than ‘Black, Asian Minority Ethnic’ children and young people.^20^ Although, notably, there is a marked lack of quality and completeness of data for many ethnic groups coupled often with small sample sizes within groups, often resulting in aggregation of ethnic groups sometimes into one all-encompassing group, and so the estimates should be considered with caution.^20^ Together these findings raise the possibility that the mental health needs of minoritised groups of children and young people are overlooked and the particular contribution of experiences of racism remains unclear, particularly given wider potential issues with access to services, recognition of poor mental health among ethnic minorities as well as the recording of these conditions.

Research with adults highlights the contribution of experiences of racism to poor mental health. A review of literature from various countries found experiences of interpersonal racism, specifically discrimination, were associated with poor mental health among adults.^21^ Furthermore, an England-based study found both interpersonal racism and perceived experiences of racism were associated with an increased risk of common mental health disorders and symptoms of psychosis among adults aged 16–74 years.^22^

Given these findings, it seems highly likely that experiences of racism will also have significant impacts on the mental health of children and young people as they are in a particularly important life stage with respect to their development of self and identity^23^ and vulnerability to the development of mental health problems.^2^ Additionally, children's lives are heavily linked to experiences with and of those around them (including peers, family members and trusted others). This cross-generational link has been found between maternal experiences of interpersonal racism, specifically discrimination, and infant cortisol level; more frequent maternal reports of discrimination predicted higher cortisol levels among Black infants.^24^

Where systematic reviews have been conducted that have demonstrated significant associations between racism and poor mental health among children and young people^2,25^ they have predominantly included studies conducted in the USA. One review, for example, identified a consistent and strong relationship between racial discrimination and negative mental health conditions such as anxiety. They also found a negative relationship between racial discrimination and positive mental health indicators, such as self-worth.^25^ However, the kinds of racism faced and its impact on mental health is likely to vary depending on the country. For example, differences in the association between racism and mental health have been found between Australia and the UK and between ethnic groups within these countries.^26^ Specifically, this study found that ethnic minority children in Australia experienced more victimisation compared with those in the UK. Furthermore, among ethnic groups in Australia, indigenous children had poorer mental health, while in the UK, Pakistani and Bangladeshi children had poorer mental health.^26^ With a lack of research focusing on the associations between racism and mental health in children and young people, and particularly the specific experiences in the UK, we set out to identify the broad existing literature on the association between experiences of racism and mental health in children and young people in the UK.

## Method

This review adhered to Dobbins’^27^ rapid review criteria. The review process included the following: Identifying the research topic and question, creating search terms to identify the literature, selecting relevant studies, extracting data and finally, analysing and summarising the results. We included studies conducted in the UK between 2000 and 2020; searches were run in December 2020. Searches were re-run in June 2022 for studies conducted in the UK between December 2020 and June 2022, this was done to ensure that the review was up to date.

Inclusion criteria were: (a) peer-reviewed publication containing original data; (b) UK-based research; (c) included examination of associations between mental health and experiences of direct or indirect racism (quantitative or qualitative); (d) inclusion of an assessment of mental health outcomes; (e) participant ages up to and including 18 years of age or (if the range went beyond 18) with a mean age of 17 years or less.

The following databases were searched: Web of Science, PsychINFO, Embase, ERIC, Scopus and Medline. Search strategies for individual databases were developed by the primary reviewer (F.T.G.) with the overarching terms being ‘UK’, ‘mental health’, ‘children’ and ‘racism’. With the help of a research librarian, these terms were expanded to encompass all potential variations and combinations of keywords within these terms (see Supplementary Appendix available at https://doi.org/10.1192/bjo.2024.836). Keywords were also identified through previously published studies on the topic.

All identified studies were uploaded to Zotero for duplication removal. The remaining studies were then uploaded to Rayyan for screening; further duplications were independently checked for and removed before screening began by F.T.G. Using Rayyan, all studies were independently screened for inclusion and exclusion according to the criteria by the lead reviewer (F.T.G.). Screening followed two steps: (a) title and abstract screen for potentially eligible studies; and (b) full text screen to confirm studies for inclusion. A subset of 30 studies was screened independently at the abstract and title stage by a second reviewer (Z.Z.) in order dually to discuss/clarify any potential issues with the inclusion and exclusion criteria. The remaining titles/abstracts were then independently screened by both reviewers to exclude any clearly irrelevant studies. Any studies considered by either reviewer potentially to contain relevant data were put forward to the full text screen. All full texts were then reviewed by the two independent reviewers (F.T.G., Z.Z.). Any disagreements on whether a study meets the study inclusion criteria were resolved through discussions with a third author (C.C.). The data were then extracted independently by both reviewers (F.T.G., Z.Z.), followed by a discussion to compare any potential differences.

## Results

### Description of included studies

**Fig. 1:**
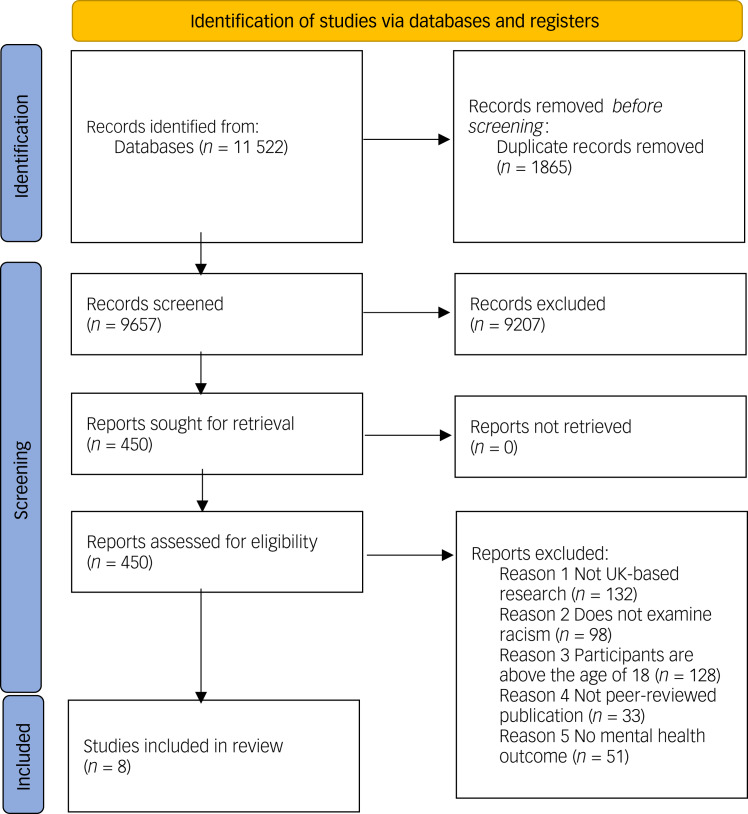
Preferred reporting items for systematic reviews and meta-analyses (PRISMA) flow diagram showing process for identifying eligible papers.

As shown in Fig. 1, in total eight studies were included in this review, with five providing quantitative data and three providing qualitative data. Details related to the study characteristics are provided in Table 2.

**Table 2:** Details of included studies

Authors, year published, title	Number of participants	Age	Race/ethnicity	Region	Approach	Measures	Environment in which racism experienced	Findings relating to the association between racism and mental health
Cassidy et al, 2004^28^ Perceived discrimination and psychological distress: the role of personal and ethnic self-esteem	154 young people	12–21 years Mean age: 17.5 years	27 Chinese, 39 Indian, 88 Pakistani	Glasgow, Scotland	Quantitative data from within a mixed methods study	Perceived discrimination (PD) scale – youth self-report HADS youth self-report	PD scale asks about experience at school and outside school	Significant direct association between perceived discrimination and anxiety among young women (*r* = 0.25, *P* < 0.05); but not depression (*r* = 0.14, ns). Perceived discrimination significantly associated with depression *r* = 0.28, *P* < 0.05) and anxiety (*r* = 0.25, *P* < 0.05). in young men.
Derrington, 2007^29^ Fight, flight and playing white: an examination of coping strategies adopted by Gypsy Traveller adolescents in English secondary schools	44 young people Number of parents and teachers not stated	11–16 years	All Gypsy traveller students	England	Qualitative data from within a mixed methods study	Interviews with students, parents and teachers	Secondary school	A parent reported that their child was having terrible mood swings, and always feeling ill in the morning. The parent believed that the child was deliberately causing himself to be ill to get out of going into school due to bullying.
Fortune et al, 2008^30^ Adolescents on preventing self-harm	6020 young people	15–16 years	4956 White, 671 Asian, 169 Black, 157 Other	Oxfordshire, Northamptonshire and Birmingham	Quantitative	Anonymous self-report, open-response questionnaire – youth self-report	Secondary school	A reference to bullying outside the home, discrimination, racism or general aggravation was made by 6% of respondents (*n* = 179). Racism as a specific form of bullying tended to be highlighted by Asian pupils, although they were less likely than the overall sample to mention bullying (4%, *n* = 15).
Astell-Burt et al, 2012^31^ Racism, ethnic density and psychological well-being through adolescence: evidence from the Determinants of Adolescent Social well-being and Health longitudinal study	6645 young people	11–16 years	White UK, Indian, Pakistani and Bangladeshi, Black Caribbean, Nigerian and Ghanaian, Other African (numbers not provided)	London	Quantitative	SDQ – youth self-report. Question specific to experiences of racism: ‘Has anyone made you feel bad or hassled you because of your race, skin colour or where you were born?’	Secondary school, home, ‘where you live’	Participants who reported having experienced racism had higher mean TDS meaning poorer psychological well-being, in comparison to those who did not, in each ethnic group. TDS mean difference (95% CI) between those who did and did not experience racism at age 12 years +1.88 (+1.75 to +2.01); +1.19 (+1.07 to +1.31) at age 16 years for boys; +2.29 (+2.12 to +2.47) at age 12 years; +1.43 (+1.29 to +1.57) at age 16 years for girls. this effect was consistent across ethnic groups.
Bécares et al, 2015^32^ A longitudinal examination of maternal, family, and area-level experiences of racism on children's socioemotional development: patterns and possible explanations	1608 mothers	Mothers of children aged 9 months to 11 years	Indian 18%, Pakistani 28%, Bangladeshi 9%, Black Caribbean 12%, Black African 14%, Other ethnic minority group 19%	UK	Quantitative data from within a mixed methods study (Millenium Cohort Study (MCS))	SDQ – parent report Harsh parent practices were measured using three items from Straus's Conflict Tactics scale Mothers were asked about experiences of received insults, disrespectful treatment from shop staff, unfair treatment, family treated unfairly, racism common.	Residential area	Support found for impact of vicarious exposure to racial discrimination experienced by mothers on child SDQ 6 years later as direct effects (coefficients range from 0.34 to 1.17) and indirect via (a) impact on maternal mental health (coefficients range from 0.004 to 0.053), and (b) impact on harsh parenting (coefficients range from 0.00 to 0.063).
McMullen et al, 2020^33^ ‘Sitting on a wobbly chair’: mental health and well-being among newcomer pupils in Northern Irish schools	116 youth workers	10–17 years	Poland, Bulgaria/Roma, Syria, Romania, Philippines, Slovakia, Hungary, Somalia, China, Thailand, Portugal, India, Lithuania, Bangladesh and Russia (numbers not mentioned)	Northern Ireland	Qualitative interviews from within a mixed methods	14 youth workers took part in focus groups (2 × 7)	Secondary school and youth centres	Both focus groups with youth workers agreed new pupils in NI faced a very real challenge of racial discrimination and that this impacts on their mental health. ‘coming out of [youth club] at night and people driving past in cars shouting insults, you know shouting things like, ‘go home’ and you know calling them [N word] and you know stuff like that.’
O'Neill et al, 2021^34^ Adolescents’ understanding of what causes emotional distress: a qualitative exploration in a non-clinical sample using ideal-type analysis	32 young people	11–12 years	White British: 22, mixed ethnic background: 4, Asian or Asian British: 2, any other White background: 3, Black or Black British: African: 1	England	Qualitative	Interviews with youth	Daily lives; school life, home life, family, friendships and feelings and emotions	Anna also believed that she was disliked and bullied at her old school because of her nationality *‘*Because I'm [states nationality]. Like, they didn't understand what being different was’. She feels that she was treated differently as a result which led to events that caused emotional distress, thus, she perceives unfairness to be the starting point of her distress.

ns, not significant; PM_2.5_ refers to minuscule particles in the air that can be harmful; SDQ, Strengths and Difficulties Questionnaire; TDS, total difficulties score; HADS, Hospital Anxiety and Depression Scale; NI, Northern Ireland.

Studies varied widely in terms of who provided the data (parents, youth workers, young people), sample size (from 14 youth workers to 6645 young people), demographic profiles, study region and the focus of the study. Only four of the studies had a specific focus on how racism affects youth mental health.^28,31,32,35^ The majority of studies (*n* = 7/8) focused on children and young people aged 10 years or over. Only one study focused on young children under the age of 10 years,^32^ and this specifically explored how racism affected the children's mothers. As shown in Table 2, seven out of eight studies focused on experiences within school settings, with five of these studies also asking about racism both within and outside of school. Four studies used youth self-report for mental health and racism, while one study used parental report and three studies used interviews (parents, youth workers and young people).

Studies providing quantitative data varied in measures of racism, with two ^31,32^ asking just one question each regarding racism (‘How common are insults or attacks to do with someone's race or colour?’; ‘Has anyone made you feel bad or hassled you because of your race, skin colour or where you were born?) and one study^35^ asking both (‘Has anyone made you feel bad or hassled you because of your race, skin colour, or where you were born?’ and ‘Has anyone made you feel bad or hassled you because of your religion?’). One study used both the Hospital Anxiety and Depression Scale (HADS) and perceived discrimination scale.^28^ The latter scale was a combination of two measures, one used by^36^ and the other developed by.^37^ Youth mental health outcome measures also varied; the Strengths and Difficulties Questionnaire (SDQ) was used in three studies^31,32,35^ and one study used an anonymous self-report open-response questionnaire, in which a reference to bullying was made.^30^

For two of the qualitative studies, relevant data were secondary to the main aims of the studies.^29,33^ They were included due to one quote in the studies that referred to the effect of racism and racism on young people's mental health. One study explored perceived causes of emotional distress among their participants and was also included due to one quote referring to the effects of racism.^34^

### Study findings

Four studies directly focused on the effects of racism on the mental health of children and young people^28,31,32,35^ and one study focused on the prevention of self-harm among adolescents.^30^ The four studies that examined youth self-reported mental health symptoms found evidence for a significant direct association between perceived discrimination, reported racism and youth mental health symptoms (anxiety among young women and depression and anxiety among young men,^28^ total difficulties in the SDQ,^31^ and increased conduct problems.^35^

Bécares et al^32^ specifically focused on maternal experiences of racism and families’ experiences of unfair treatment, and reported a significant indirect association between maternal experiences and parent reported SDQ scores, which was mediated by self-reported ‘harsh parenting’. Fortune et al^30^ focused on self-harm prevention and found that 6% of the respondents referred to discrimination and racism, and although Asian pupils, compared with the overall sample, were less likely to mention bullying, they tended to highlight racism as a specific form of bullying. The remaining three studies provided qualitative data, although there was just one relevant quote from each that related to how racism affected the mental health of children and young people. Two of these quotes were given by a parent and youth worker, and only one was from a young person.

## Discussion

While we identified both quantitative and qualitative data that highlight the potential negative impacts of experiences of racism on young people, the most striking finding from this review is the lack of available literature on the potential mental health impacts of racism on young people in the UK. The lack of research in this area is telling and could reflect a lack of prioritisation in both research and funding on this issue. Only eight studies met our inclusion criteria, and the effects of racism on children and young people's mental health was an incidental finding in four of these studies. Furthermore, three of these studies provided little information on the specific nature of experiences and contexts that might create particular risks for youth mental health and how they should best be addressed. This includes a lack of consideration of how experiences may vary across ethnic and cultural groups among other identity traits. These are important limitations given that studies have shown that experiences of racism vary widely for people within the broad ‘Black, Asian and Minority Ethnic’ category,^38^ and within ethnic groups, factors such as sex and gender may be associated with different experiences, often referred to as intersectionality. These findings highlight significant gaps that ought to be addressed in future research.

The vast majority of studies that were identified during the searches were conducted outside of the UK, mainly in the USA. Other studies that focused on experiences of racism in the UK did not specifically explore potential effects on participants’ mental health nor provide specific data on those under 18 years.^39,40^ In addition to the overall lack of studies, there was also a lack of consistency in approaches taken to assess both experiences of racism and youth mental health symptoms, and whether the mental health measures used are appropriate across ethnicities and cultures remains unclear.^41^ The majority of studies also specifically focused on experiences within secondary school settings, particularly experiences of interpersonal racism.

Notably, no studies examined the impact of internalised racism despite a systematic review indicating that internalised racism has been linked to low levels of self-esteem, lower career aspirations, increased feelings of hopelessness and stress, as well as psychological conditions such as depression, anxiety and body dissatisfaction.^42^

Furthermore, no studies examined the impact of experiences relating to structural and institutional racism despite young people stressing the impact these experiences can have on their identity and sense of belonging.^43,44^ As an example, a recurrent negative experience recounted by young people in the UK is police stop and search,^45^ which has both interpersonal and institutional elements of racism. While the interaction occurs between individuals (a suspect and an officer), when these individual instances are aggregated, disparities emerge along racial lines, suggesting structural or institutional factors. For instance, ‘Black’ children in England and Wales are 6.5 times more likely to be subjected by police to strip searching, a particularly intrusive form of stop and search, than ‘White’ children.^46^ ‘Black’ children are also more likely than ‘Asian’ and ‘Mixed’ children to be strip searched.^46^ Furthermore, young people describe code switching, when an individual will adjust their self-presentation in order to receive outcomes they deem desirable,^47^ in response to a breakdown in trust between communities and institutions.^44^ For example, young people may feel pressured to adjust their self-presentation, or ‘perform,’ in order to protect themselves from, and appear more palatable to, those who might otherwise negatively perceive them. Young people have highlighted that these kinds of behaviours form part of the training and preparation they have been given by their parents, potentially indicating indirect effects of racism and the intergenerational transmission of racial trauma.^44,48^

One study identified in our review highlighted parental experiences of racism as having a potential indirect effect on the mental health of children under the age of 10 years.^32^ In this case, the focus was on maternal experiences of racism and how this affects children's socioemotional development.^32^ Indeed, looking at the wider literature, a systematic review of the impacts of vicarious racism found a significant association with children's health in almost half of the studies.^2^ Commonly, the categories of health studies were birth outcomes, socioemotional and mental health. Notably, just over half of the studies failed to find a significant association between vicarious racism and child health. However, this may be due to several factors, such as measurement and methodological variability. Heard-Garris et al^2^ commented on the need for more studies exploring the relationship between child health and vicarious racism given that children's exposure to racism can occur in various ways, such as through experiences of peers, caregivers and through the media.

Strengths of this study include our adherence to guidelines for rapid reviews; however, restricting our searches to peer-reviewed studies means we may have excluded research published in other formats and our findings have a risk of publication bias. Given the diverse approaches used, we did not conduct quality ratings for individual studies. Our focus on the UK was deliberate; it inevitably reduces generalisation to other settings but also highlights the need for specific consideration of similar questions in other countries where knowledge may be equally limited.

The findings from this review underline the urgent need for research to understand further the mental health impacts of racism on children and young people, and the moderators and mediators of their effects in order ultimately to guide the (co-)development of effective, culturally and developmentally appropriate interventions to promote good mental health and to treat and prevent mental health problems. Going forward, there is a clear need for validated measures of experiences of racism that are appropriate across cultures and are available in multiple languages to promote consistency in measurement across studies. In one meta-analysis, nine different exposure instruments were used to measure self-reported racism across 138 studies.^9^ Critically, future research should also be underpinned by anti-racism research principles to ensure it increases awareness and addresses the issues^49^ without unintentionally bringing harm.

A number of research priorities arise from this review. First, there is a clear need for more extensive robust evaluation of the association between racism and the mental health of children and young people in the UK. In particular, there should be exploration of the impacts of vicarious, systemic and institutional racism, and there should be particular emphasis on children under the age of 10 years. It will also be imperative to take into account intersectional factors such as sex and gender, as well as a consideration of differences within minority ethnic groups, avoiding the pitfalls associated with the use of terms such as ‘BAME’ which group together people and communities that may have had disparate experiences. Despite the small number of studies identified, the breadth of measurement approaches, operationalisation of racism and methods of categorising individuals (e.g. on the basis of race, ethnicity and ancestry) is striking. Ongoing research would benefit from the development of a universal framework for measuring experiences of racism in order to improve consistency of measurement and reporting across studies.

## Supporting information

Ghezae et al. supplementary material Ghezae et al. supplementary material

## Data Availability

Data availability is not applicable to this article as no new data were created or analysed in this study.
