# Supplementary material for: The association between experiences of racism and mental health on children and young people in the UK: rapid scoping review
Source: BJPsych Open. 2025 Jan 27;11(1):e26. doi: 10.1192/bjo.2024.836 (PMC11822989; doi:10.1192/bjo.2024.836)
Supplement: Ghezae et al. supplementary material [file S2056472424008366sup001.docx]

|  | **Scopus** |
| --- | --- |
| UK | ( TITLE-ABS-KEY ( "united kingdom"  OR  "national health service"  OR  nhs  OR  gb  OR  "g.b."  OR  britain*  OR  uk  OR  "u.k."  OR  "united kingdom*"  OR  england*  OR  british*  OR  "northern ireland*"  OR  "northern irish*"  OR  scotland*  OR  scottish*  OR  wales  OR  welsh* ) ) |
| Mental health | ( TITLE-ABS-KEY ( "mental health"  OR  "mental disorder*"  OR  depressi*  OR  anxi*  OR  psychos*  OR  psychot*  OR  "mood disorder*"  OR  "eating disorder*"  OR  "mental illness" ) ) |
| Children | ( ( TITLE-ABS-KEY ( infan*  OR  newborn*  OR  new-born *  OR  perinat*  OR  neonat*  OR  baby*  OR  babies  OR  toddler* OR  minor*  OR  boy*  OR  girl*  OR  kid  OR  kids  OR  child*  OR schoolchild*  OR  adolescen*  OR  juvenil* OR  youth*  OR  teen*  OR  pubescen*  OR  pediatric*  OR  paediatric*  OR  peadiatric* ) OR  TITLE-ABS-KEY ( school*  OR  prematur*  OR  preterm*  OR  "pre term*"  OR  prepub*  OR  pre-pub*  OR  preschool*  OR pre-school*  OR  kindergarten*  OR  nursery*  OR  preadolescen*  OR  pre-adolescen* ) ) ) |
| Racism | ( ( TITLE-ABS-KEY ( "BAME"  OR  bme  OR  ( "Black Asian"  W/1  "minority ethnic" )  OR  "minority ethnic*"  OR  "ethnic minorit*"  OR  ( racial  W/5  disparit* )  OR  ( ethnic  W/5  disparit* )  OR  "people of color"  OR  "people of colour"  OR  poc  OR  "racial* minorit*"  OR  "Race Factor*"  OR  "mixed race" )  OR  TITLE-ABS-KEY ( "mixed racial"  OR  minorit*  OR  "ethnic* group*" )  AND  ( "Black British"  OR  bangladeshi*  OR  "indian subcontinent"  OR  bengali*  OR  indian*  OR  chinese  OR  pakistani*  OR  african*  OR  gyps*  OR "irish traveller*"  OR  roma  OR  arab*  OR  "afro caribbean" )  OR  TITLE-ABS-KEY ( "african caribbean"  OR  afrocaribbean  OR  "afro-caribbean"  OR  "south asian*"  OR  refugee*  OR  migrant*  OR  immigrant*  OR  asylum  AND seeker*  OR  jew* )  OR  TITLE-ABS-KEY ( racism  OR  prejudice*  OR  racial*  OR  segregat*  OR  unfair*  OR  microaggressi*  OR  "micro aggressi*"  OR  racist* ) ) ) |

|  | **Medline** |
| --- | --- |
| UK | 1 exp United Kingdom/  2 (national health service* or nhs*).ti,ab,in.  3 (english not ((published or publication* or translat* or written or language* or speak* or literature or citation*) adj5 english)).ti,ab.  4 (gb or "g.b." or britain* or (british* not "british columbia") or uk or "u.k." or united kingdom* or (england* not "new england") or northern ireland* or northern irish* or scotland* or scottish* or ((wales or "south wales") not "new south wales") or welsh*).ti,ab,jw,in.  5 (bath or "bath's" or ((birmingham not alabama*) or ("birmingham's" not alabama*) or bradford or "bradford's" or brighton or "brighton's" or bristol or "bristol's" or carlisle* or "carlisle's" or (cambridge not (massachusetts* or boston* or harvard*)) or ("cambridge's" not (massachusetts* or boston* or harvard*)) or (canterbury not zealand*) or ("canterbury's" not zealand*) or chelmsford or "chelmsford's" or chester or "chester's" or chichester or "chichester's" or coventry or "coventry's" or derby or "derby's" or (durham not (carolina* or nc)) or ("durham's" not (carolina* or nc)) or ely or "ely's" or exeter or "exeter's" or gloucester or "gloucester's" or hereford or "hereford's" or hull or "hull's" or lancaster or "lancaster's" or leeds* or leicester or "leicester's" or (lincoln not nebraska*) or ("lincoln's" not nebraska*) or (liverpool not (new south wales* or nsw)) or ("liverpool's" not (new south wales* or nsw)) or ((london not (ontario* or ont or toronto*)) or ("london's" not (ontario* or ont or toronto*)) or manchester or "manchester's" or (newcastle not (new south wales* or nsw)) or ("newcastle's" not (new south wales* or nsw)) or norwich or "norwich's" or nottingham or "nottingham's" or oxford or "oxford's" or peterborough or "peterborough's" or plymouth or "plymouth's" or portsmouth or "portsmouth's" or preston or "preston's" or ripon or "ripon's" or salford or "salford's" or salisbury or "salisbury's" or sheffield or "sheffield's" or southampton or "southampton's" or st albans or stoke or "stoke's" or sunderland or "sunderland's" or truro or "truro's" or wakefield or "wakefield's" or wells or westminster or "westminster's" or winchester or "winchester's" or wolverhampton or "wolverhampton's" or (worcester not (massachusetts* or boston* or harvard*)) or ("worcester's" not (massachusetts* or boston* or harvard*)) or (york not ("new york*" or ny or ontario* or ont or toronto*)) or ("york's" not ("new york*" or ny or ontario* or ont or toronto*))))).ti,ab,in.  6 (bangor or "bangor's" or cardiff or "cardiff's" or newport or "newport's" or st asaph or "st asaph's" or st davids or swansea or "swansea's").ti,ab,in.   1. (aberdeen or "aberdeen's" or dundee or "dundee's" or edinburgh or "edinburgh's" or glasgow or "glasgow's" or inverness or (perth not australia*) or ("perth's" not australia*) or stirling or "stirling's").ti,ab,in. 2. (armagh or "armagh's" or belfast or "belfast's" or lisburn or "lisburn's" or londonderry or "londonderry's" or derry or "derry's" or newry or "newry's").ti,ab,in.   9 1 or 2 or 3 or 4 or 5 or 6 or 7 or 8  10 (exp africa/ or exp americas/ or exp antarctic regions/ or exp arctic regions/ or exp asia/ or exp australia/ or exp oceania/) not (exp United Kingdom/ or europe/)  11 9 not 10 |
| Mental Health | 12 Mental Health/  13 exp Mental Disorders/  14 ("mental health" or "mental disorder*" or depressi* or anxi* or psychos* or psychot* or "mood disorder*" or "eating disorder*" or "mental illness").ti,ab.  15 12 or 13 or 14 |
| Children | 16 adolescent/ or exp child/ or exp infant/  17 (infan* or newborn* or new-born* or perinat* or neonat* or baby* or babies or toddler* or minor* or boy* or girl* or kid or kids or child* or schoolchild* or adolescen* or juvenil* or youth* or teen* or pubescen* or pediatric* or paediatric* or peadiatric* or school* or prematur* or preterm* or "pre term*" or prepub* or pre-pub* or preschool* or pre-school* or kindergarten* or nursery* or preadolescen* or pre-adolescen*).ti,ab.  18 16 or 17 |
| Racism | 19 Racism/  20 Race Relations/  21 Prejudice/  22 exp Ethnic Groups/  23 Minority Groups/  24 (("BAME" or BME or ("Black Asian" adj1 "minority ethnic") or "minority ethnic*" or "ethnic minorit*" or (racial adj5 disparit*) or (ethnic adj5 disparit*) or "people of color" or "people of colour" or POC or "racial* minorit*" or "Race Factor*" or "mixed race" or "mixed racial" or minorit* or "ethnic* group*") and ("Black British" or bangladeshi* or "indian subcontinent" or bengali* or indian* or chinese or pakistani* or african* or gyps* or "irish traveller*" or roma or arab* or "afro caribbean" or "african caribbean" or afrocaribbean or "afro-caribbean" or "south asian*" or Refugee* or migrant* or Immigrant* or asylum seeker* or Jew*)).ti,ab.   1. (racism or prejudice* or racial* or segregat* or unfair* or microaggressi* or "micro aggressi*" or racist*).ti,ab. 2. 19 or 20 or 21 or 22 or 23 or 24 or 25 |

|  | **Embase** |
| --- | --- |
| UK | 1 exp United Kingdom/  2 (national health service* or nhs*).ti,ab,in.  3 (english not ((published or publication* or translat* or written or language* or speak* or literature or citation*) adj5 english)).ti,ab.  4 (gb or "g.b." or britain* or (british* not "british columbia") or uk or "u.k." or united kingdom* or (england* not "new england") or northern ireland* or northern irish* or scotland* or scottish* or ((wales or "south wales") not "new south wales") or welsh*).ti,ab,jw,in.  5 (bath or "bath's" or ((birmingham not alabama*) or ("birmingham's" not alabama*) or bradford or "bradford's" or brighton or "brighton's" or bristol or "bristol's" or carlisle* or "carlisle's" or (cambridge not (massachusetts* or boston* or harvard*)) or ("cambridge's" not (massachusetts* or boston* or harvard*)) or (canterbury not zealand*) or ("canterbury's" not zealand*) or chelmsford or "chelmsford's" or chester or "chester's" or chichester or "chichester's" or coventry or "coventry's" or derby or "derby's" or (durham not (carolina* or nc)) or ("durham's" not (carolina* or nc)) or ely or "ely's" or exeter or "exeter's" or gloucester or "gloucester's" or hereford or "hereford's" or hull or "hull's" or lancaster or "lancaster's" or leeds* or leicester or "leicester's" or (lincoln not nebraska*) or ("lincoln's" not nebraska*) or (liverpool not (new south wales* or nsw)) or ("liverpool's" not (new south wales* or nsw)) or ((london not (ontario* or ont or toronto*)) or ("london's" not (ontario* or ont or toronto*)) or manchester or "manchester's" or (newcastle not (new south wales* or nsw)) or ("newcastle's" not (new south wales* or nsw)) or norwich or "norwich's" or nottingham or "nottingham's" or oxford or "oxford's" or peterborough or "peterborough's" or plymouth or "plymouth's" or portsmouth or "portsmouth's" or preston or "preston's" or ripon or "ripon's" or salford or "salford's" or salisbury or "salisbury's" or sheffield or "sheffield's" or southampton or "southampton's" or st albans or stoke or "stoke's" or sunderland or "sunderland's" or truro or "truro's" or wakefield or "wakefield's" or wells or westminster or "westminster's" or winchester or "winchester's" or wolverhampton or "wolverhampton's" or (worcester not (massachusetts* or boston* or harvard*)) or ("worcester's" not (massachusetts* or boston* or harvard*)) or (york not ("new york*" or ny or ontario* or ont or toronto*)) or ("york's" not ("new york*" or ny or ontario* or ont or toronto*))))).ti,ab,in.  6 (bangor or "bangor's" or cardiff or "cardiff's" or newport or "newport's" or st asaph or "st asaph's" or st davids or swansea or "swansea's").ti,ab,in.   1. (aberdeen or "aberdeen's" or dundee or "dundee's" or edinburgh or "edinburgh's" or glasgow or "glasgow's" or inverness or (perth not australia*) or ("perth's" not australia*) or stirling or "stirling's").ti,ab,in. 2. (armagh or "armagh's" or belfast or "belfast's" or lisburn or "lisburn's" or londonderry or "londonderry's" or derry or "derry's" or newry or "newry's").ti,ab,in.   9 1 or 2 or 3 or 4 or 5 or 6 or 7 or 8  10 (exp africa/ or exp americas/ or exp antarctic regions/ or exp arctic regions/ or exp asia/ or exp australia/ or exp oceania/) not (exp United Kingdom/ or europe/)  11 #9 not #10 |
| Mental Health | 12 Mental Health/  13 exp Mental Diseases/  14 ("mental health" or "mental disorder*" or depressi* or anxi* or psychos* or psychot* or "mood disorder*" or "eating disorder*" or "mental illness").ti,ab.  15 12 or 13 or 14 |
| Children | 16 adolescent/ or exp child/ or exp infant/  17 (infan* or newborn* or new-born* or perinat* or neonat* or baby* or babies or toddler* or minor* or boy* or girl* or kid or kids or child* or schoolchild* or adolescen* or juvenil* or youth* or teen* or pubescen* or pediatric* or paediatric* or peadiatric* or school* or prematur* or preterm* or "pre term*" or prepub* or pre-pub* or preschool* or pre-school* or kindergarten* or nursery* or preadolescen* or pre-adolescen*).ti,ab.  18 16 or 17 |
| Racism | 19 Racism/  20 Race Relations/  21 Prejudice/  22 exp Ethnic Groups/  23 Minority Groups/  24 (("BAME" or BME or ("Black Asian" adj1 "minority ethnic") or "minority ethnic*" or "ethnic minorit*" or (racial adj5 disparit*) or (ethnic adj5 disparit*) or "people of color" or "people of colour" or POC or "racial* minorit*" or "Race Factor*" or "mixed race" or "mixed racial" or minorit* or "ethnic* group*") and ("Black British" or bangladeshi* or "indian subcontinent" or bengali* or indian* or chinese or pakistani* or african* or gyps* or "irish traveller*" or roma or arab* or "afro caribbean" or "african caribbean" or afrocaribbean or "afro-caribbean" or "south asian*" or Refugee* or migrant* or Immigrant* or asylum seeker* or Jew*)).ti,ab.   1. (racism or prejudice* or racial* or segregat* or unfair* or microaggressi* or "micro aggressi*" or racist*).ti,ab. 2. 19 or 20 or 21 or 22 or 23 or 24 or 25 |

|  | **PsychINFO** |
| --- | --- |
| UK | 1 exp United Kingdom/  2 (national health service* or nhs*).ti,ab,in.  3 (english not ((published or publication* or translat* or written or language* or speak* or literature or citation*) adj5 english)).ti,ab.  4 (gb or "g.b." or britain* or (british* not "british columbia") or uk or "u.k." or united kingdom* or (england* not "new england") or northern ireland* or northern irish* or scotland* or scottish* or ((wales or "south wales") not "new south wales") or welsh*).ti,ab,jw,in.  5 (bath or "bath's" or ((birmingham not alabama*) or ("birmingham's" not alabama*) or bradford or "bradford's" or brighton or "brighton's" or bristol or "bristol's" or carlisle* or "carlisle's" or (cambridge not (massachusetts* or boston* or harvard*)) or ("cambridge's" not (massachusetts* or boston* or harvard*)) or (canterbury not zealand*) or ("canterbury's" not zealand*) or chelmsford or "chelmsford's" or chester or "chester's" or chichester or "chichester's" or coventry or "coventry's" or derby or "derby's" or (durham not (carolina* or nc)) or ("durham's" not (carolina* or nc)) or ely or "ely's" or exeter or "exeter's" or gloucester or "gloucester's" or hereford or "hereford's" or hull or "hull's" or lancaster or "lancaster's" or leeds* or leicester or "leicester's" or (lincoln not nebraska*) or ("lincoln's" not nebraska*) or (liverpool not (new south wales* or nsw)) or ("liverpool's" not (new south wales* or nsw)) or ((london not (ontario* or ont or toronto*)) or ("london's" not (ontario* or ont or toronto*)) or manchester or "manchester's" or (newcastle not (new south wales* or nsw)) or ("newcastle's" not (new south wales* or nsw)) or norwich or "norwich's" or nottingham or "nottingham's" or oxford or "oxford's" or peterborough or "peterborough's" or plymouth or "plymouth's" or portsmouth or "portsmouth's" or preston or "preston's" or ripon or "ripon's" or salford or "salford's" or salisbury or "salisbury's" or sheffield or "sheffield's" or southampton or "southampton's" or st albans or stoke or "stoke's" or sunderland or "sunderland's" or truro or "truro's" or wakefield or "wakefield's" or wells or westminster or "westminster's" or winchester or "winchester's" or wolverhampton or "wolverhampton's" or (worcester not (massachusetts* or boston* or harvard*)) or ("worcester's" not (massachusetts* or boston* or harvard*)) or (york not ("new york*" or ny or ontario* or ont or toronto*)) or ("york's" not ("new york*" or ny or ontario* or ont or toronto*))))).ti,ab,in.  6 (bangor or "bangor's" or cardiff or "cardiff's" or newport or "newport's" or st asaph or "st asaph's" or st davids or swansea or "swansea's").ti,ab,in.   1. (aberdeen or "aberdeen's" or dundee or "dundee's" or edinburgh or "edinburgh's" or glasgow or "glasgow's" or inverness or (perth not australia*) or ("perth's" not australia*) or stirling or "stirling's").ti,ab,in. 2. (armagh or "armagh's" or belfast or "belfast's" or lisburn or "lisburn's" or londonderry or "londonderry's" or derry or "derry's" or newry or "newry's").ti,ab,in.   9 1 or 2 or 3 or 4 or 5 or 6 or 7 or 8  10 ((Africa or america or antartctic region or artic reigons or asia or australia or oceania) not (United Kingdom or europe)).mp. [mp=title, abstract, heading word, table of contents, key concepts, original title, tests & measures, mesh]  11 #9 not #10 |
| Mental Health | 12 Mental Health/  13 exp Mental Diseases/  14 ("mental health" or "mental disorder*" or depressi* or anxi* or psychos* or psychot* or "mood disorder*" or "eating disorder*" or "mental illness").ti,ab.  15 12 or 13 or 14 |
| Children | 1. Adolescent 2. exp Infant Development/ or exp Sudden Infant Death/ or exp "Bayley Scales of Infant Development"/ or exp Infant Vocalization/ or exp Infant Temperament/ or infant.mp.   18 exp Child Self Care/ or exp Child Care Workers/ or exp Father Child Relations/ or exp Child Abuse Reporting/ or exp Child Psychiatry/ or exp Child Abuse/ or exp Child Psychology/ or exp Parent Child Relations/ or exp Child Neglect/ or exp Father Child Communication/ or exp Mother Child Communication/ or exp Mother Child Relations/ or exp Child Health/ or exp Child Psychotherapy/ or exp Child Welfare/ or exp Child Day Care/ or child.mp. or exp Child Death/ or exp Child Support/ or exp "Adoption (Child)"/ or exp Child Visitation/ or exp Child Guidance/ or exp Parent Child Communication/ or exp Child Care/ or exp Child Attitudes/ or exp Child Custody/ or exp Child Discipline/ or exp Child Labor/ or exp Child Characteristics/ or exp Child Behavior/ or exp Child Psychopathology/ or exp Child Behavior Checklist/  19 (infan* or newborn* or new-born* or perinat* or neonat* or baby* or babies or toddler* or minor* or boy* or girl* or kid or kids or child* or schoolchild* or adolescen* or juvenil* or youth* or teen* or pubescen* or pediatric* or paediatric* or peadiatric* or school* or prematur* or preterm* or "pre term*" or prepub* or pre-pub* or preschool* or pre-school* or kindergarten* or nursery* or preadolescen* or pre-adolescen*).ti,ab  20 16 or 17 or 18 or 19 |
| Racism | 21 Racism/  22 Race Relations/  23 Prejudice/  24 exp Ethnic Groups/  25 Minority Groups/  26 (("BAME" or BME or ("Black Asian" adj1 "minority ethnic") or "minority ethnic*" or "ethnic minorit*" or (racial adj5 disparit*) or (ethnic adj5 disparit*) or "people of color" or "people of colour" or POC or "racial* minorit*" or "Race Factor*" or "mixed race" or "mixed racial" or minorit* or "ethnic* group*") and ("Black British" or bangladeshi* or "indian subcontinent" or bengali* or indian* or chinese or pakistani* or african* or gyps* or "irish traveller*" or roma or arab* or "afro caribbean" or "african caribbean" or afrocaribbean or "afro-caribbean" or "south asian*" or Refugee* or migrant* or Immigrant* or asylum seeker* or Jew*)).ti,ab.  27 (racism or prejudice* or racial* or segregat* or unfair* or microaggressi* or "micro aggressi*" or racist*).ti,ab.  28 21 or 22 or 23 or 24 or 25 or 26 or 27 |

|  | **Webofscience** |
| --- | --- |
| UK | 1 CU=(United Kingdom)   1. (TI=(national health service* or nhs*) OR AB=(national health service* or nhs*)) 2. TI=(english not ((published or publication* or translat* or written or language* or speak* or literature or citation*) NEAR5 english)) OR AB=(english not ((published or publication* or translat* or written or language* or speak* or literature or citation*) NEAR5 english)) 3. (TI=(gb or "g.b." or britain* or (british* not "british columbia") or uk or "u.k." or united kingdom* or (england* not "new england") or northern ireland* or northern irish* or scotland* or scottish* or ((wales or "south wales") not "new south wales") or welsh*) or AB=(gb or "g.b." or britain* or (british* not "british columbia") or uk or "u.k." or united kingdom* or (england* not "new england") or northern ireland* or northern irish* or scotland* or scottish* or ((wales or "south wales") not "new south wales") or welsh*)) 4. (TI=(bath or "bath's" or ((birmingham not alabama*) or ("birmingham's" not alabama*) or bradford or "bradford's" or brighton or "brighton's" or bristol or "bristol's" or carlisle* or "carlisle's" or (cambridge not (massachusetts* or boston* or harvard*) ) or ("cambridge's" not (massachusetts* or boston* or harvard*) ) or (canterbury not zealand*) or ("canterbury's" not zealand*) or chelmsford or "chelmsford's" or chester or "chester's" or chichester or "chichester's" or coventry or "coventry's" or derby or "derby's" or (durham not (carolina* or nc) ) or ("durham's" not (carolina* or nc) ) or ely or "ely's" or exeter or "exeter's" or gloucester or "gloucester's" or hereford or "hereford's" or hull or "hull's" or lancaster or "lancaster's" or leeds* or leicester or "leicester's" or (lincoln not nebraska*) or ("lincoln's" not nebraska*) or (liverpool not (new south wales* or nsw) ) or ("liverpool's" not (new south wales* or nsw) ) or ((london not (ontario* or ont or toronto*) ) or ("london's" not (ontario* or ont or toronto*) ) or manchester or "manchester's" or (newcastle not (new south wales* or nsw) ) or ("newcastle's" not (new south wales* or nsw) ) or norwich or "norwich's" or nottingham or "nottingham's" or oxford or "oxford's" or peterborough or "peterborough's" or plymouth or "plymouth's" or portsmouth or "portsmouth's" or preston or "preston's" or ripon or "ripon's" or salford or "salford's" or salisbury or "salisbury's" or sheffield or "sheffield's" or southampton or "southampton's" or st albans or stoke or "stoke's" or sunderland or "sunderland's" or truro or "truro's" or wakefield or "wakefield's" or wells or westminster or "westminster's" or winchester or "winchester's" or wolverhampton or "wolverhampton's" or (worcester not (massachusetts* or boston* or harvard*) ) or ("worcester's" not (massachusetts* or boston* or harvard*) ) or (york not ("new york*" or ny or ontario* or ont or toronto*) ) or ("york's" not ("new york*" or ny or ontario* or ont or toronto*) )))) OR AB=(bath or "bath's" or ((birmingham not alabama*) or ("birmingham's" not alabama*) or bradford or "bradford's" or brighton or "brighton's" or bristol or "bristol's" or carlisle* or "carlisle's" or (cambridge not (massachusetts* or boston* or harvard*) ) or ("cambridge's" not (massachusetts* or boston* or harvard*) ) or (canterbury not zealand*) or ("canterbury's" not zealand*) or chelmsford or "chelmsford's" or chester or "chester's" or chichester or "chichester's" or coventry or "coventry's" or derby or "derby's" or (durham not (carolina* or nc) ) or ("durham's" not (carolina* or nc) ) or ely or "ely's" or exeter or "exeter's" or gloucester or "gloucester's" or hereford or "hereford's" or hull or "hull's" or lancaster or "lancaster's" or leeds* or leicester or "leicester's" or (lincoln not nebraska*) or ("lincoln's" not nebraska*) or (liverpool not (new south wales* or nsw) ) or ("liverpool's" not (new south wales* or nsw) ) or ((london not (ontario* or ont or toronto*) ) or ("london's" not (ontario* or ont or toronto*) ) or manchester or "manchester's" or (newcastle not (new south wales* or nsw) ) or ("newcastle's" not (new south wales* or nsw) ) or norwich or "norwich's" or nottingham or "nottingham's" or oxford or "oxford's" or peterborough or "peterborough's" or plymouth or "plymouth's" or portsmouth or "portsmouth's" or preston or "preston's" or ripon or "ripon's" or salford or "salford's" or salisbury or "salisbury's" or sheffield or "sheffield's" or southampton or "southampton's" or st albans or stoke or "stoke's" or sunderland or "sunderland's" or truro or "truro's" or wakefield or "wakefield's" or wells or westminster or "westminster's" or winchester or "winchester's" or wolverhampton or "wolverhampton's" or (worcester not (massachusetts* or boston* or harvard*) ) or ("worcester's" not (massachusetts* or boston* or harvard*) ) or (york not ("new york*" or ny or ontario* or ont or toronto*) ) or ("york's" not ("new york*" or ny or ontario* or ont or toronto*) ))))) 5. TI=(bangor or "bangor's" or cardiff or "cardiff's" or newport or "newport's" or st asaph or "st asaph's" or st davids or swansea or "swansea's") OR AB=(bangor or "bangor's" or cardiff or "cardiff's" or newport or "newport's" or st asaph or "st asaph's" or st davids or swansea or "swansea's") 6. TI=(aberdeen or "aberdeen's" or dundee or "dundee's" or edinburgh or "edinburgh's" or glasgow or "glasgow's" or inverness or (perth not australia*) or ("perth's" not australia*) or stirling or "stirling's") OR AB=(aberdeen or "aberdeen's" or dundee or "dundee's" or edinburgh or "edinburgh's" or glasgow or "glasgow's" or inverness or (perth not australia*) or ("perth's" not australia*) or stirling or "stirling's") 7. TI=(armagh or "armagh's" or belfast or "belfast's" or lisburn or "lisburn's" or londonderry or "londonderry's" or derry or "derry's" or newry or "newry's") OR AB=(armagh or "armagh's" or belfast or "belfast's" or lisburn or "lisburn's" or londonderry or "londonderry's" or derry or "derry's" or newry or "newry's") 8. #8 OR #7 OR #6 OR #5 OR #4 OR #3 OR #2 OR #1   10 ALL=( africa* OR americas* OR antarctic* regions OR arctic* regions OR asia* OR australia* OR oceania*) NOT ALL=( United Kingdom* OR europe*)  11 #9 not #10 |
| Mental Health | 12 ALL= Mental Health/   1. ALL= Mental Diseases/ 2. TI=("mental health" or "mental disorder*" or depressi* or anxi* or psychos* or psychot* or "mood disorder*" or "eating disorder*" or "mental illness") OR AB=("mental health" or "mental disorder*" or depressi* or anxi* or psychos* or psychot* or "mood disorder*" or "eating disorder*" or "mental illness")   15 #14 OR #13 OR #12 |
| Children | 1. ALL=(adolescent OR child OR infant) 2. TI=(infan* or newborn* or new-born* or perinat* or neonat* or baby* or babies or toddler* or minor* or boy* or girl* or kid or kids or child* or schoolchild* or adolescen* or juvenil* or youth* or teen* or pubescen* or pediatric* or paediatric* or peadiatric* or school* or prematur* or preterm* or "pre term*" or prepub* or pre-pub* or preschool* or pre-school* or kindergarten* or nursery* or preadolescen* or pre-adolescen*) OR AB=(infan* or newborn* or new-born* or perinat* or neonat* or baby* or babies or toddler* or minor* or boy* or girl* or kid or kids or child* or schoolchild* or adolescen* or juvenil* or youth* or teen* or pubescen* or pediatric* or paediatric* or peadiatric* or school* or prematur* or preterm* or "pre term*" or prepub* or pre-pub* or preschool* or pre-school* or kindergarten* or nursery* or preadolescen* or pre-adolescen*) 3. #17 OR #16 |
| Racism | 19 ALL= Racism/  20 ALL= Race Relations/  21 ALL= Prejudice/  22 ALL= Ethnic Groups/  23 ALL= Minority Groups/  24 TI=(("BAME" or BME or ("Black Asian" NEAR1 "minority ethnic") or "minority ethnic*" or "ethnic minorit*" or (racial NEAR5 disparit*) or (ethnic NEAR5 disparit*) or "people of color" or "people of colour" or POC or "racial* minorit*" or "Race Factor*" or "mixed race" or "mixed racial" or minorit* or "ethnic* group*") and ("Black British" or bangladeshi* or "indian subcontinent" or bengali* or indian* or chinese or pakistani* or african* or gyps* or "irish traveller*" or roma or arab* or "afro caribbean" or "african caribbean" or afrocaribbean or "afro-caribbean" or "south asian*" or Refugee* or migrant* or Immigrant* or asylum seeker* or Jew*) ) OR AB=(("BAME" or BME or ("Black Asian" NEAR1 "minority ethnic") or "minority ethnic*" or "ethnic minorit*" or (racial NEAR5 disparit*) or (ethnic NEAR5 disparit*) or "people of color" or "people of colour" or POC or "racial* minorit*" or "Race Factor*" or "mixed race" or "mixed racial" or minorit* or "ethnic* group*") and ("Black British" or bangladeshi* or "indian subcontinent" or bengali* or indian* or chinese or pakistani* or african* or gyps* or "irish traveller*" or roma or arab* or "afro caribbean" or "african caribbean" or afrocaribbean or "afro-caribbean" or "south asian*" or Refugee* or migrant* or Immigrant* or asylum seeker* or Jew*) )   1. TI=(racism or prejudice* or racial* or segregat* or unfair* or microaggressi* or "micro aggressi*" or racist*) OR AB=(racism or prejudice* or racial* or segregat* or unfair* or microaggressi* or "micro aggressi*" or racist*) 2. #25 OR #24 OR #23 OR #22 OR #21 OR #20 OR #19 |

|  | **ERIC** |
| --- | --- |
| UK | 1. United Kingdom 2. national health service* or nhs* 3. (english not ((published or publication* or translat* or written or language* or speak* or literature or citation*) N5 english)) 4. (gb or "g.b." or britain* or (british* not "british columbia") or uk or "u.k." or united kingdom* or (england* not "new england") or northern ireland* or northern irish* or scotland* or scottish* or ((wales or "south wales") not "new south wales") or welsh*) 5. (bath or "bath's" or ((birmingham not alabama*) or ("birmingham's" not alabama*) or bradford or "bradford's" or brighton or "brighton's" or bristol or "bristol's" or carlisle* or "carlisle's" or (cambridge not (massachusetts* or boston* or harvard*)) or ("cambridge's" not (massachusetts* or boston* or harvard*)) or (canterbury not zealand*) or ("canterbury's" not zealand*) or chelmsford or "chelmsford's" or chester or "chester's" or chichester or "chichester's" or coventry or "coventry's" or derby or "derby's" or (durham not (carolina* or nc)) or ("durham's" not (carolina* or nc)) or ely or "ely's" or exeter or "exeter's" or gloucester or "gloucester's" or hereford or "hereford's" or hull or "hull's" or lancaster or "lancaster's" or leeds* or leicester or "leicester's" or (lincoln not nebraska*) or ("lincoln's" not nebraska*) or (liverpool not (new south wales* or nsw)) or ("liverpool's" not (new south wales* or nsw)) or ((london not (ontario* or ont or toronto*)) or ("london's" not (ontario* or ont or toronto*)) or manchester or "manchester's" or (newcastle not (new south wales* or nsw)) or ("newcastle's" not (new south wales* or nsw)) or norwich or "norwich's" or nottingham or "nottingham's" or oxford or "oxford's" or peterborough or "peterborough's" or plymouth or "plymouth's" or portsmouth or "portsmouth's" or preston or "preston's" or ripon or "ripon's" or salford or "salford's" or salisbury or "salisbury's" or sheffield or "sheffield's" or southampton or "southampton's" or st albans or stoke or "stoke's" or sunderland or "sunderland's" or truro or "truro's" or wakefield or "wakefield's" or wells or westminster or "westminster's" or winchester or "winchester's" or wolverhampton or "wolverhampton's" or (worcester not (massachusetts* or boston* or harvard*)) or ("worcester's" not (massachusetts* or boston* or harvard*)) or (york not ("new york*" or ny or ontario* or ont or toronto*)) or ("york's" not ("new york*" or ny or ontario* or ont or toronto*))))) 6. (bangor or "bangor's" or cardiff or "cardiff's" or newport or "newport's" or st asaph or "st asaph's" or st davids or swansea or "swansea's") 7. (aberdeen or "aberdeen's" or dundee or "dundee's" or edinburgh or "edinburgh's" or glasgow or "glasgow's" or inverness or (perth not australia*) or ("perth's" not australia*) or stirling or "stirling's") 8. (armagh or "armagh's" or belfast or "belfast's" or lisburn or "lisburn's" or londonderry or "londonderry's" or derry or "derry's" or newry or "newry's") 9. S1 OR S2 OR S3 OR S4 OR S5 OR S6 OR S7 OR S8 10. (africa/ or americas/ or antarctic regions/ or arctic regions/ or asia/ or australia/ or oceania/) not ( United Kingdom/ or europe/) 11. S9 NOT S10 |
| Mental Health | 1. Mental Health/ 2. Mental Disorders/ 3. ("mental health" or "mental disorder*" or depressi* or anxi* or psychos* or psychot* or "mood disorder*" or "eating disorder*" or "mental illness") 4. S12 OR S13 OR S14 |
| Children | 1. adolescent OR child OR infant 2. (infan* or newborn* or new-born* or perinat* or neonat* or baby* or babies or toddler* or minor* or boy* or girl* or kid or kids or child* or schoolchild* or adolescen* or juvenil* or youth* or teen* or pubescen* or pediatric* or paediatric* or peadiatric* or school* or prematur* or preterm* or "pre term*" or prepub* or pre-pub* or preschool* or pre-school* or kindergarten* or nursery* or preadolescen* or pre-adolescen*) 3. S16 OR S17 |
| Racism | 19 Racism  20 Race Relations  21 Prejudice  22 Ethnic Groups  23 Minority Groups   1. (("BAME" or BME or ("Black Asian" N1 "minority ethnic") or "minority ethnic*" or "ethnic minorit*" or (racial N5 disparit*) or (ethnic N5 disparit*) or "people of color" or "people of colour" or POC or "racial* minorit*" or "Race Factor*" or "mixed race" or "mixed racial" or minorit* or "ethnic* group*") and ("Black British" or bangladeshi* or "indian subcontinent" or bengali* or indian* or chinese or pakistani* or african* or gyps* or "irish traveller*" or roma or arab* or "afro caribbean" or "african caribbean" or afrocaribbean or "afro-caribbean" or "south asian*" or Refugee* or migrant* or Immigrant* or asylum seeker* or Jew*)) 2. (racism or prejudice* or racial* or segregat* or unfair* or microaggressi* or "micro aggressi*" or racist*)   26 S19 OR S20 OR S21 OR S22 OR S23 OR S24 OR S25 |
